# Myonecrosis in a Sickle Cell-Diseased Patient: A Case Report and Literature Review

**DOI:** 10.1155/2020/3189645

**Published:** 2020-08-18

**Authors:** Ali Wasel Almomen, Yaser Mansour Tawfeeq, Ahmed Khalid Almuhaisin, Abdulraheem Ayman Altalib

**Affiliations:** ^1^King Fahad Specialist Hospital Dammam, Orthopedic Surgery, Saudi Arabia; ^2^Imam Abdulrahman Bin Faisal University King Fahd Hospital of the University, Orthopedic Surgery, Saudi Arabia

## Abstract

Myonecrosis is a condition that results in muscle tissue necrosis, and it is rarely seen in sickle cell patients and often missed because more common manifestations of sickle cell can overlap like vaso-occlusive crises (VOC), which results in pain in the extremities or spine; also osteomyelitis is commonly seen in sickle cell patients. In this article, we present a case of myonecrosis in a sickle cell-diseased patient who presented with left acute atraumatic left foot pain and MRI with contrast showing characteristic image of muscle infarction supportive therapy initiated, and 2-year follow-up did not reveal any physical disabilities; further study and follow-up are needed to know the nature of the disease and avoid improper management of those patients.

## 1. Introduction

Sickle cell disease (SCD) is the most common hematological inheritable condition worldwide [1]. The prevalence of SCD in the eastern province of Saudi Arabia is 145 cases/10,000 persons, which is the highest in the kingdom [2]. It is characterized by a single missense mutation in the beta-globin gene and can present as homozygous hemoglobin S (HbSS) or compound heterozygous hemoglobin S. When tension is lowered, hemoglobin becomes distorted and rigid and binds to postcapillary venules, which leads to vascular trapping and reduced blood flow, precipitating vaso-occlusive crisis (VOC), which can result in myonecrosis [3]. Myonecrosis in sickle cell patients is rare and is, therefore, frequently missed, partly because of its rarity in sickle cell patients, and in part to the scarcity of literature on this specific topic (to date, only 15 cases have been reported) [3]. Another reason that myonecrosis may be missed is that its signs and symptoms could be mistaken for VOC or osteomyelitis, both of which are common in SCD.

## 2. Case Presentation

Herein, we present a case of a 30-year-old Saudi Arabian gentleman known to have SCD (HBSS) and complaining of sudden onset right-foot pain for 3 days. The pain was continuous and nonradiating. There was no history of fever, trauma, or a similar prior episode. With respect to the patient's SCD history, he had had no major complications of SCD, with an average number of hospital admissions of one or two per year for VOC pain management. He had no history of previous intensive care unit (ICU) admissions, surgical interventions, or blood transfusions, and he had no current drug use.

**Figure 1:**
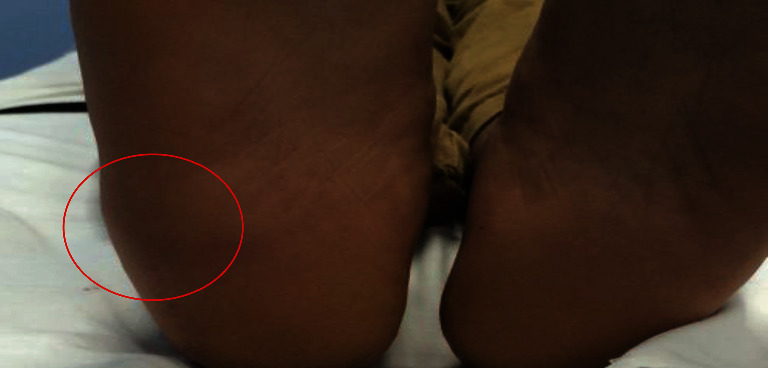
Clinical photograph with red circle showing swelling in the right foot.

On physical examination, the patient's vital signs were within the normal range. The patient had an antalgic gait and full active and passive range of movement with minimal pain. The right foot showed a swelling 4 × 6 cm^2^, 3 cm below the lateral malleolus, extending to the planter aspect of the foot. The swelling was firm, warm, and mildly tender with no signs of erythema, discharge, or fluctuation. Blood vessels were palpable (Figure 1).

Laboratory investigations revealed the following findings: complete blood count (CBC): WBC: 11.5\L, hemoglobin (Hgb): 12.0 g\dl, and platelet (PLT): 149,000\L. Renal panel: normal. liver panel: normal. Lactic acid dehydrogenase (LDH), creatine kinase (CK), and CK-MB: normal. Erythrocyte sedimentation rate (ESR) = 20. C-reactive protein (CRP): negative. Alkaline phosphatase: normal.

**Figure 2:**
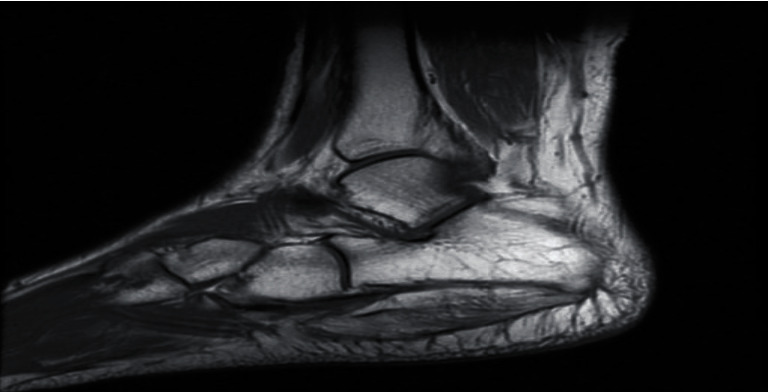
Magnetic resonance image of the right foot, Sag T1 showing diffuse enlargement of the muscle, with increased signal intensity. The muscle fibers are intact.

**Figure 3:**
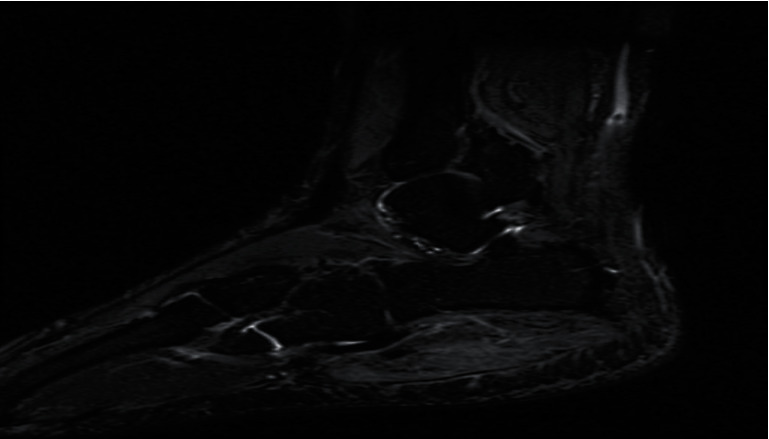
Short T1 inverse recovery (STIR) showing enlargement of the muscle with diffuse heterogeneous increase in signal intensity, with no drop of signal on fat-saturated image and normal signal intensity of the bone.

**Figure 4:**
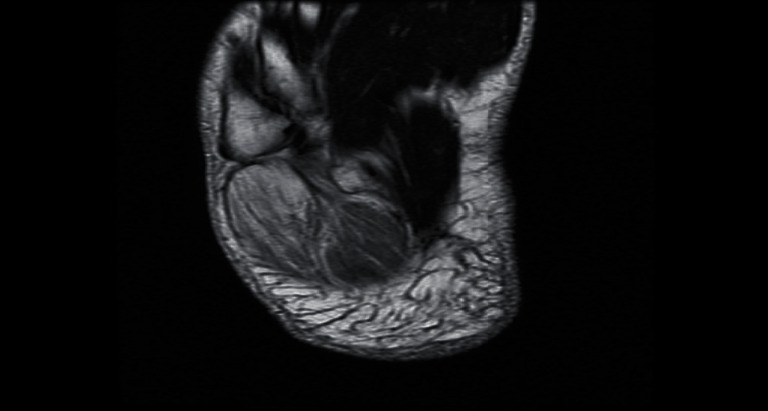
Axial T2 showing abnormally high T2 signal intensity along the muscle fibers with involvement of the adjacent muscle (flexor digitorum brevis).

**Figure 5:**
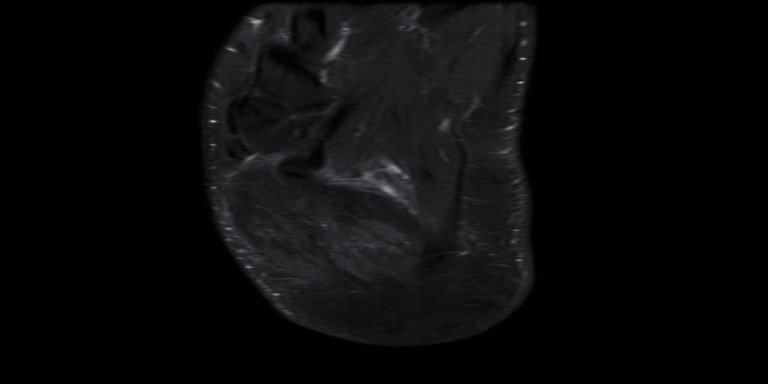
Axial T1 images post contrast demonstrating ill-defined heterogeneous enhancement of the abductor digiti minimi muscle, with no wall (ring) enhancement.

Magnetic resonance imaging (MRI) with contrast showed increased signal intensity in T1 and T2 of the abductor digiti minimi and flexor digitorum brevis, diffuse muscular edema in short T1 inverse recovery (STIR), and heterogeneous enhancement of post gadolinium with no wall enhancement. There were no bony changes. This MRI reading together with the clinical picture was indicative of acute muscle infarction, indicative of myonecrosis (Figures 2–5).

The patient was managed with intravenous (IV) fluid hydration and NSAIDS and was advised not to bear weight on his right lower limb. In addition, he underwent extensive physiotherapy in the form of a range of motion and strengthening exercises of the left foot and ankle.

The patient was followed up after 2 weeks at 6 months, 1 year, and 2 years. The pain and swelling had resolved completely after two weeks. The 6-month outpatient follow-up also showed that he was pain-free with no complaints and no gross abnormality or deformity of the right foot and ankle.

## 3. Discussion

While the exact pathophysiology of myonecrosis in SCD is unknown, it is most likely due to the rigidity of the red blood cells, which, in turn, causes a decrease in blood supply to the muscle, causing ischemia. This tissue ischemia and hypoxia cause an inflammatory process that leads to muscular edema that can cause muscular infarction, which in turn leads to myofibrosis [12].

Myonecrosis in SCD patients is commonly missed probably because of the following two reasons: first, the lack of experience of the primary care providers with such cases; and second, the common association of SCD with VOC and infections. It is important to note that the latter reason will require the patient to undergo unnecessary treatment [13].

Up until January 2017, there have been 15 cases of sickle cell myonecrosis (including ours) reported (Table 1). Most patients were young adults, with a mean age of 28 years (with the exception of one patient, who was a child). The male to female ratio was 2 : 1. All the patients had HbSS disease with a history of previously treated pain crises [3].

Painful swelling is the most common presentation of myonecrosis. There have been no reports of fever. Ten of the reported patients had bilateral muscle involvement with the proximal large muscle group, usually involving the lower limbs, which are most commonly involved in a ratio of 2 : 1. The involvement of the joints is usually in the form of decreases in range of motion due to pain.

Misdiagnosis of myonecrosis will lead to a delay in treatment or leave the disease untreated, which will cause progressive necrosis of myocytes, leading to muscle atrophy, fibrosis, and contractures. Finally, liquefactive necrosis can occur, resulting in the formation of a sterile abscess. This abscess is typically nonpurulent on drainage [4, 5]. Other atypical features include the development of compartment syndrome (which necessitated fasciotomies in 2 of the reported patients) [6, 7].

Laboratory investigation, while not useful for determining a specific diagnosis of myonecrosis, may be useful to rule out a differential diagnosis such as infection. CPK and LDH were reported to be high in 4 and 5 patients, respectively; however, all remaining patients showed normal levels of CPK and LDH, making these unreliable parameters for the diagnosis of myonecrosis [3].

Muscle biopsy is the gold standard for diagnosing myonecrosis; however, if the MRI and clinical findings are conclusive of a diagnosis of myonecrosis, biopsy is not indicated [5]. The necrosis of myocytes causes alteration in muscle size and shape, which increases signal intensity on T2-weighed images and gadolinium enhancement, as shown by MRI [8].

Intravenous nonsteroidal anti-inflammatory drugs (NSAIDS) have been reported as being sufficient for pain control. Aggressive physiotherapy in the form of nonweight bearing, pain-relieving modalities, and range of motion exercises to prevent fibrosis, which may cause contracture and stiffness of an involved joint, may be suggested as treatment options [3]. Swelling usually subsides within weeks, and the patient should be followed up for functional assessment of the affected limb.

Clear guidelines for treating myonecrosis have not yet been established; however, an early and accurate diagnosis and treatment, in addition to physical therapy, are essential for preventing complications such as contractures, fibrosis, and muscle atrophy [3].

## 4. Conclusion

Although myonecrosis is a well-known complication of SCD, treating physicians may not be aware of this as a possible diagnosis; therefore, it may frequently be missed, delaying necessary treatment. Moreover, further follow and study may be needed in order to help physicians to identify myonecrosis more easily.

## Figures and Tables

**Table 1:** Reported cases of myonecrosis in sickle cell patients.

Age (years)	Sex	Findings	CPK^∗^ (IU/L)	LDH⋀ (IU/L)	Muscles involved	Reference
30	M	Pain, swelling, warmth, tenderness	300	—	B/L deltoids, B/L quadriceps	2
22	M	Pain, swelling, warmth, tenderness	—	—	B/L deltoids, B/L thighs	2
26	F	Pain, swelling, warmth, tenderness	300	—	B/L thigh and knee	3
4	M	Pain, swelling, tenderness	—	—	Right thigh and knee	3
44	F	Pain, swelling, indurated contracture	—	—	B/L deltoids	4
36	M	Pain, swelling, indurated contracture	—	—	B/L quadriceps	4
26	M	Pain, swelling induration	—	—	B/L quadriceps	4
30	M	Pain, swelling indurated contracture	—	—	B/L thighs	5
42	F	Induration	Normal	—	Left elbow	6
24	M	Pain, swelling, warmth, erythema	2140	592	Left leg	7
24	F	Pain, swelling, warmth, erythema	644	—	B/L thighs	8
28	M	Pain, swelling, warmth	185	1164	Left thigh	9
28	F	Pain, swelling, induration	624	431	B/L deltoids, B/L triceps	10
27	M	Pain, swelling, erythema	—	689	Plantar muscles	11
30	M	Pain, swelling, warmth	—	—	Plantar muscles	(our case)

Abbreviations: CPK: Creatine Phospho-Kinase; ⋀ LDH: Lactate Dehydrogenase, B/L: bilateral.

## References

[1] Hankins J. S., Wang W. C., Greer J. P., Foerster J., Rodgers G. M. (2008). Sickle cell anemia and other sickling syndromes. *Wintrobe’s Clinical Hematology*.

[2] Alotaibi M. M. (2017). Sickle cell disease in Saudi Arabia: a challenge or not. *Journal of Epidemiology and Global Health*.

[3] Tageja N., Racovan M., Valent J., Zonder J. (2010). Myonecrosis in Sickle Cell Anemia—Overlooked and Underdiagnosed. *Case Reports in Medicine*.

[4] Malekgoudarzi B., Feffer S. (1999). Myonecrosis in sickle cell anemia. *The New England Journal of Medicine*.

[5] Groves J., Stiles R. G. (1997). Sickle cell myonecrosis involving the plantar musculature. *Journal of the American Podiatric Medical Association*.

[6] Schumacher H. R., Murray W. M., Dalinka M. K. (1990). Acute muscle injury complicating sickle cell crisis. *Seminars in Arthritis and Rheumatism*.

[7] May D. A., Disler D. G., Jones E. A., Balkissoon A. A., Manaster B. J. (2000). Abnormal signal intensity in skeletal muscle at MR imaging: patterns, pearls, and pitfalls. *Radiographics*.

[8] Turaga L. P., Boddu P., Kipferl S., Basu A., Yorath M. (2017). Myonecrosis in sickle cell anemia: case study. *American Journal of Case Reports*.

[9] Ejindu V. C., Hine A. L., Mashayekhi M., Shorvon P. J., Misra R. R. (2007). Musculoskeletal manifestations of sickle cell disease. *Radiographics*.

[10] Dorwart B. B., Gabuzda T. G. (1985). Symmetric myositis and fasciitis: a complication of sickle cell anemia during vasoocclusion. *The Journal of Rheumatology*.

[11] Valeriano-Marcet J., Kerr L. D. (1991). Myonecrosis and myofibrosis as complications of sickle cell anemia. *Annals of Internal Medicine*.

[12] Dennis G. J., Keating R. M. (1991). Muscle infarction in sickle cell anemia. *Annals of Internal Medicine*.

[13] Mani S., Duffy T. P. (1993). Sickle myonecrosis revisited. *The American Journal of Medicine*.

[14] Vicari P., Achkar R., Oliveira K. R. B. (2004). Myonecrosis in sickle cell anemia: case report and review of the literature. *Southern Medical Journal*.

[15] Rubio M. Á., Díez L., Álvarez N., Munteis E. (2015). Afectacion muscular en la anemia drepanocitica. *Medicina Clínica (English Edition)*.

